# Communication of Pharmacogenomic test results and treatment plans in pediatric oncology: deliberative stakeholder consultations with parents

**DOI:** 10.1186/s12904-021-00709-2

**Published:** 2021-01-12

**Authors:** Cristina Longo, Vasiliki Rahimzadeh, Gillian Bartlett

**Affiliations:** 1 Department of Family Medicine, McGill University, 5858 Chemin de la Côtes-des-Neiges, Suite 300, Montreal, Quebec, H3S 1Z1 Canada; 2 Department of Respiratory Medicine, Amsterdam University Medical Center, Location Academic Medical Center, Meibergdreef 9, Office F5-259, Amsterdam, North Holland 1105AZ the Netherlands; 3 Stanford Center for Biomedical Ethics, 1215 Welch Rd Modular A, Stanford, CA 94305 USA

**Keywords:** Pharmacogenetic testing, Communication, Brain cancer, Diagnosis, Pediatrics

## Abstract

**Background:**

Effective communication in support of clinical decision-making is central to the pediatric cancer care experience for families. A new laboratory derived pharmacogenetic test (LDT) that can diagnose difficult-to-treat brain cancers has been developed to stratify children based on their ability to respond to available treatment; however, the potential implementation of the LDT may make effective communication challenging since it can potentially remove the option for curative treatment in those children identified as non-responders, i.e. those with a catastrophic diagnosis.

**Objective:**

We solicited the perspectives of parents of children with difficult-to-treat brain cancer on communication preferences surrounding the potential implementation of the LDT in standard care using deliberative stakeholder consultations.

**Methods:**

Eight bereaved parents of children who succumbed to difficult-to-treat brain cancer, and four parents of children currently undergoing treatment for similar cancers attended separate small-group deliberative consultations – a stakeholder engagement method that enables the co-creation of recommendations following the consideration of competing arguments and diverse opinions of parents with different experiences. In the small-group consultations (Phase I), parents discussed four questions about potential communication issues that may arise with the LDT in practice. In Phase II, a total of five parents from both stakeholder groups (4 bereaved and 1 in current treatment) attended a consultation, known as the ‘mixed’ consultation, with the purpose of co-developing concrete recommendations for implementation of the LDT.

**Results:**

Explaining the risks, benefits, and accuracy of the LDT were considered essential to parents. Once an LDT-based diagnosis/prognosis can be made, parents valued honesty, empathy, and clarity in communication. Parents also requested that all results and treatment options be presented to them in measured doses, and in an unbiased manner over the course of several meetings. This communication strategy allowed sufficient time to understand and accept the diagnosis/prognosis, particularly if it was catastrophic. Continuous access to the appropriate psychological and social support or counselling at and post-diagnosis was also strongly recommended.

**Conclusions:**

Deliberants co-created family-centered recommendations surrounding communication issues of the LDT, providing guidance to pediatric oncologists that could implement the test in practice.

**Supplementary Information:**

The online version contains supplementary material available at 10.1186/s12904-021-00709-2.

## Background

Effective communication in pediatric oncology is a crucial component of the overall care experience and fostering a supportive, honest, and trusting environment for shared decision-making [1]. Nevertheless, delivering bad news, whether it is the diagnosis of cancer itself or test results indicating a poor prognosis, is one of the most challenging aspects of practicing oncology. This is because communication skills are not systematically taught as part of most standard residency training or continuing medical education programs [1, 2]. In the pediatric oncology setting, distinct issues arise in the communication between physician, parent, and child. Despite fighting their own emotions regarding the unnatural threat to a child’s life [3], the clinical team must not only determine the best manner in which to deliver bad news to the family, but also navigate the complex tripartite decision-making process, where applicable [1]. Moreover, oncologists must achieve a delicate balance when disclosing poor, yet honest and clear prognoses, while also taking care not to remove all hope [4].

With the growing implementation of pharmacogenomic tests that stratify children with cancer by expected treatment outcome and risk, effective communication is becoming increasingly difficult for pediatric oncologists. For example, a laboratory-derived blood test (LDT) that can accurately identify fatal genetic mutations in ~ 20% children with difficult-to-treat brain cancers (i.e. high-grade glioblastomas, HGGs) is being considered for implementation as a standard of care [5, 6]. For children carrying these fatal mutations, palliative care would be introduced at diagnosis because these tumors have been shown to be treatment-refractory [5, 6]. Nevertheless, treatments known to be ineffective may still be frequently administered to children despite serious side-effects that reduce quality of life without clinical benefit; this could stem from the physicians’ difficulty in communicating or the parents’ inability to accept an end-of-life prognosis as well as the need to maintain hope for a recovery [7, 8]. The clinical decision to pursue comfort, rather than curative therapies for children with fatal or ‘catastrophic’ HGG diagnoses, can be distressing for the pediatric oncologist and the family. Emotional coping strategies, such as anger and disbelief at the limited treatment options, may challenge effective communication and shared decision-making in these devastating cases.

Patient engagement methods can identify implementation barriers for practice-change interventions, such as the LDT [9]. One such method, deliberative stakeholder consultations, promotes active debate surrounding a health policy issue of interest, such as LDT-based communication issues, while weighing the competing arguments of other stakeholders. Its ultimate goal is to arrive at recommendations that can aid in decision-making [9, 10]. The deliberative element, i.e. the refining of opinions from the consideration of competing arguments to achieve consensus and/or co-develop recommendations, distinguishes this method from others such as focus groups and structured interviews [11].

As our goal was to develop family-centered recommendations for communicating genetic test results and treatment options, with an emphasis on breaking the news of a catastrophic diagnosis, we used deliberative stakeholder consultations to solicit the perspectives of parents of children with HGG on the use of this potential LDT. We specifically explored what information parents deem necessary to understand the nature of the diagnosis, how results should be communicated, and their preferences with regard to the available therapy options. The recommendations generated from this study could then provide guidance to pediatric oncologists when these challenging scenarios arise.

## Methods

### Research design and recruitment

We conducted small-group deliberative consultations with two stakeholder groups and future users of the LDT: 1) bereaved parents of children diagnosed with HGG, and 2) parents of children currently undergoing treatment for brain cancer, including but not limited to HGG. We selected bereaved parents as they are the experts in receiving the news of a catastrophic diagnosis. Parents currently navigating the diagnostic uncertainties related to their child’s brain cancer, regardless of their prognosis, were also selected as the LDT would be administered to all children with suspected brain cancer. Parents were identified and purposefully recruited via email with the help of Meagan’s Walk, a community-based organization supporting families affected by childhood brain cancer located in Toronto, Ontario. This study was approved by McGill University’s Institutional Review Board.

### Informing parents: details regarding the genetic test for HGG treatment decision-making

At the beginning of each small-group consultation, the facilitator (GB) explained the goals of the deliberative process to parents. These goals included proposing and raising competing arguments as well as soliciting diverse opinions from interested stakeholders. The expert also obtained informed consent at the start of each deliberation to record the sessions. GB then provided a lay summary of the following regarding the non-invasive LDT: 1) the accuracy by which the test identifies fatal mutations from biomarkers in blood (and is therefore less invasive than a brain tumor biopsy); 2) the survival statistics of those children carrying the fatal mutations (e.g. H3.3 K27M and H3.1 K27M) despite having received conventional treatment (i.e. chemotherapy) based on previous research; 3) the logistics of the genetic test (e.g. the test will be part of the initial diagnostic workup and rapid turnaround time for results); 4) the proposed course of action based on the test results; and 5) benefit-risk considerations relating to chemotherapy and/or radiation as well as its potential impact on the child’s quality of life.

### Consultation design and data collection

**Fig. 1:**
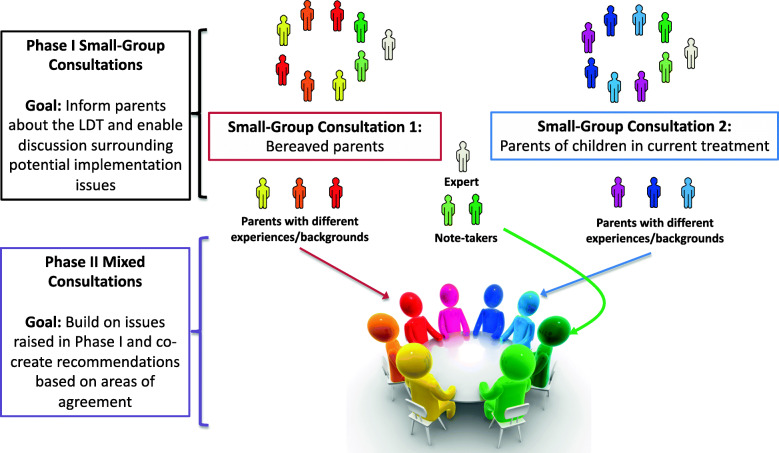
Overview of the Deliberative Stakeholder Consultation Design. In Phase I, bereaved parents and parents of children with brain cancer currently in treatment attended separate 2–3 h small-group consultations, with the goal of identifying salient communication issues associated with the potential implementation of the LDT in practice. All participants in Phase I were subsequently invited to attend the Phase II mixed consultation, where parents co-created family-centered recommendations regarding the pediatric oncology team’s LDT-based communication approach

Our overall consultation design has been previously described in detail elsewhere (Fig. 1) [10]. Briefly, bereaved parents and those with children currently undergoing treatment attended separate 2 to 3 h small-group consultations (Phase I). These groups attended separate consultations to encourage discussion among parents having experienced similar processes/outcomes, hypothesizing that the issues discussed and the arguments raised would be different between groups. In this initial phase, participants first listened to a lay presentation of the policy issue by the expert and were then given the opportunity to ask questions or clarifications. Following this initial information session, parents were then asked to discuss their opinions and preferences with one another relating to the implementation of the LDT in standard care. Four questions guided this discussion and are presented in Table 1. The expert only intervened to answer clarifying questions about the LDT once the deliberative process began so as to minimize undue influence on participant discussions.

**Table 1:** Consultations with parents and deliberation questions

Consultation Group	Deliberation Questions
Phase I	
Small-group consultations 1) Bereaved parents 2) Parents of children undergoing treatment	1. What information do you think families would need to understand the results of the genomic test and the implications for treatment? 2. What concerns/problems do you think families might have in accepting treatment plans based on the genomic test? 3. What members of the health care team do you think would need to be involved in discussing the test results with children and families? i.e. the oncologist, the palliative care physician, etc.? 4. Are there any other issues you can think of that may be a problem if this test was part of regular clinical care for children with brain tumors?
Phase II	
Mixed consultation - Bereaved parents and parents of children undergoing treatment	1. What and how do you think test results and treatment options should be communicated to the parents by the health care team in the initial and subsequent follow-up meetings (if different)? 2. Who do you think should be involved in the initial diagnostic consultation and subsequent meetings about potential treatment options, i.e. clinical and psychosocial support? 3. Would your recommendations from Q1) and Q2) change if the test results indicated a de-escalation of care/therapy? What margin of error do you think would make you accept the test-based diagnosis as “fact”? In other words, how certain is certain?

In Phase II, all parents from the Phase I small-group consultations were invited to attend a combined ‘mixed’ consultation. In contrast to Phase I, the goal of the Phase II mixed consultation was to co-develop family-centered recommendations regarding the communication of the LDT results in the pediatric oncology care context based on areas of consensus corroborated by the deliberations. In this mixed session, the expert first presented key themes arising from phase I consultations, which shaped the modified deliberation questions (Table 1) and were then used to guide the development of recommendations regarding the pediatric oncology team’s LDT-based communication approach.

All consultations were audio recorded and transcribed verbatim. Authors CL and VR produced detailed ethnographic reports on the interactions among participants as well as points of deliberative agreement and disagreement for each phase. All authors discussed and refined key themes that emerged from qualitative analyses of the transcripts.

### Data analysis and deliberative outputs

The audio transcripts from the small-group consultations were first analyzed using inductive thematic content analysis, focusing on identifying salient issues related to communicating LDT-based results and treatment options throughout the deliberations. Two separate coders agreed on the coding scheme and analyzed the data (CL and VR). The audio transcripts were then re-analyzed and the coders assessed whether disagreements or agreements were reached among participants during each small-group consultation, with the help of ethnographic reports, as well as the recommendations developed in the mixed consultation [12]. A summary highlighting key themes and conclusive collective statements made by the parents, outlining agreement, disagreement, or a recommendation for future course of action, were considered to be the primary outputs in the small-group and mixed consultations, respectively. Summary documents were submitted to and ratified by participants following each session. We also assessed the quality of the deliberations via administration of two surveys evaluating deliberative quality criteria described by De Vries and colleagues following each consultation (Online Supplement) [13]. The quality of the deliberations was considered to be sufficient if participants reported high ratings of the deliberative process, i.e. feeling listened to, respected, procedural fairness, and helpfulness of the information presented.

## Results

### Overview of themes and deliberants

In the separate small-group consultations, 8 were parents who were bereaved and 4 were parents of children currently undergoing treatment; in the mixed consultation, 5 parents (4 bereaved and 1 currently in treatment) accepted the invitation to participate. In the bereaved parents consultation, the discussions centered around important physician-parent communication values, such as honesty and empathy, providing families with the appropriate psychological and social support or counselling at and post-diagnosis, as well as providing parents with unbiased information to enable shared decision-making of the child’s treatment trajectory. In addition to these themes, the parents of children currently undergoing treatment deliberated on communication strategy details, i.e. the structure and content of physician-family meetings, as well as the inclusion of the child in such conversations. We first present the initial themes arising from both Phase I small-group consultations, as there was significant overlap between groups (see Table 2 for an overview of themes and quotes). We then present the six concrete family-centered recommendations for LDT-based communication in practice co-developed by the parents in the mixed consultation. We also note that, based on the deliberation quality assessment results, all consultations were considered to be of high-quality (Online Supplement).

**Table 2:** Overview of Topics and Themes Discussed in the Phase I Small-Group Deliberative Consultations

Timing of Communication	Topic	Themes	Parents Quotes
At the initial diagnostic encounter (whether catastrophic diagnosis or not)	Information on the LDT	Benefits/risks Accuracy Treatment implications	*“But then on top of accuracy, I think you would want to know … as a parent, if you’re getting the treatment, what is your child going to choose? What are the chances of curing it? Is it worth it?”* *“If the [HGG] can be diagnosed in a blood test, that kind of takes out that whole … You know, is any parent going to agree to a biopsy? We were told it was a pretty high risk; she could have come out of it paralyzed.”*
	Delivering the diagnosis	Honesty, empathy, and compassion Use of medical jargon and clarity	*“I asked if [my child] was going to die, and [the surgeon] looked at me and said, well everyone is going to die someday.”* *“It’s that human side of the doctors, and still that professional side of having to deliver the information.”* *“But thank goodness, most of them are honest, and that’s what we needed. And I think that’s important for physicians and oncologists to know that.”* *“I had no idea what [the diagnosis] was […*] *I didn’t even know [my child] had cancer until they presented us with this clinical trial and as my mom was reading the drugs, she’s like … well that’s chemotherapy. And then, I’m like … [my child] has cancer?”* *“I kept telling people, oh no, my son doesn’t have cancer. It’s just a low grade glioma. He doesn’t have cancer […] and then the Canadian Cancer society sent us a letter saying [my child] is registered as a cancer patient. Oh okay – maybe it’s cancer then […] but I haven’t heard [cancer] from a doctor. [...] The [doctors] try to help us to cope, you know. But sometimes it’s better you give us some straight information.”*
	Support Personnel	Emotional support Communication support Genetic Counselling	*“You need someone who knows what has been happening […] almost like a bereaved parent, who can guide you through it.”* *“Maybe it’s the social worker or someone else who comes in to take notes […] because you’re listening but you’re not really listening. As soon as the diagnosis comes in, it’s like bla bla bla, the teacher from Charlie Brown.”* *“If you can test for this, then I want my other children tested … because if it’s in the DNA, they would see that as a whole family thing as I can imagine […] I would just sit there and think … is this going to happen to my other child?”* *“I’m sure it’s in the back of [my other son’s] mind … what if this is genetic and I have it?”*
Once a catastrophic diagnosis is confirmed (either at initial diagnosis or at a relapse/treatment failure)	Communicating bad news and acceptance of diagnosis	Validating prognosis with second opinions Evidence supporting prognosis Multiple meetings with repetition of information Maintaining hope Coordinated message among clinical team members Continuity of care and trust building	*“So I think, if it’s something that they’re saying, “Yes, this is certain,” I think they should be prepared for parents to say, “What is that second opinion? I need to hear this from somebody else, and maybe even from a third person, before I’m going to accept this.” Because we, even though they gave us the worst news, we looked for alternative medicines and all sorts of different things online, just like anybody else I’m sure. But that’s the process that any parent, I think, is going to go through; so they have to prepare those answers […*]” *“And have the backup of … “It’s not just this result; we’ve also got the MRI. We’ve also got …*” *“If you have more pieces [of information] that you can put together later when you’re thinking about the [encounter] then it’s a little bit easier.”* *“It took a while to register. It took at least 4 meetings with [our doctor] before it got into our heads that there is nothing [they can do].”* *“If your child is in the 1% that doesn’t respond to anything, you’re taking away their hope. And the human spirit and hope is a huge factor. If you believe in something and have faith that something is going to work, sometimes [it works].”* *“One doctor came in and said, we’ve got bad news, the drug didn’t work and you guys are going to go into radiation. Then he left and another doctor comes in and says, oh we have wonderful news, we’re going to start the radiation soon. One doctor is telling you one thing, and the other says wonderful news. Which one is it?”* *“I mean, that’s one of the biggest problems too, right, is that you’re always seeing a different doctor […] And if one of them brought this new test, to give this … And you didn’t know that doctor, and you hadn’t seen them much before, and you didn’t quite like them […] to trust that this test was useful or that you would want to do it […] But they all seem to work that way now. You don’t see the same doctor. It’s always somebody else.”*
	Palliative care team Introduction	Timing of introduction	*“It’s almost like they were all there to protect themselves from us freaking out on them. I don’t know. I felt a little up against the wall with so many people being there delivering that news.”*
Once the diagnosis is understood and accepted	Communicating Treatment Options	Honesty Unbiased and clear information	*“We needed someone to say to us that chances are, even if it does work, [your child] will probably not last another year … So how do you want that year to go? A lot depends on what the physicians are telling you.”* *“It’s going to be tough for the physicians to give the right “answer” [regarding treatment options]. They gave us both sides of the coin […] Ultimately, it’s a chance of a chance, but you as parents are the ones that have to make that decision. And I think that’s tough for a lot of parents to hear. Even though they are not the professional, they will still have to make that choice at some point. Our care was amazing […] it was always that honest, up front, there isn’t hope but we’ll help you if you want to look at other things … We’ll help you if you want to do these trials, but we’re also here to make [your child’s] life as comfortable as possible for what we think is left of it.”*
Meeting following the presentation of treatment options	Treatment Decision-Making	Quality of life as a primary concern Involving the child in decision-making	*“The worst part was chemotherapy. […] At that time you think you’re doing the right thing, because that’s what they are telling you to do […] They’re sick, then they’re skinny, then they’re fat, then they’re skinny, then they’re fat. Thinking back on it now, I wish I would have just taken him home and enjoyed my time with him.”* *“You have your discussion with the doctor and then bring [your child] in after and say,[…] we’ve been discussing different [treatment] options and what we think is best for you is this. And what do you think? Do you have any questions?”*

### Phase I small-group deliberative consultations

Parents first discussed how the LDT would be introduced at initial presentation. The accuracy, risks, benefits (non-invasiveness compared to biopsy), turn-around time, and implications of the test concerning response to treatment were seen as key communication needs. At initial presentation, bereaved parents expressed frustration with the health care team when asked questions that implied their parenting skills or the environment in which they lived were the potential causes of the brain tumor:

*“One nurse asked me, did you breast feed?” (BP8)*

*“Did [your child] ever hit his head?” (BP3)*

Once a diagnosis and prognosis can be communicated to the family, all parents suggested that oncologists “be human” when delivering the news, expecting a balance of honesty, compassion, and empathy. One participant remembers:

*“I asked if [my child] was going to die, and [the surgeon] looked at me and said, well everyone is going to die someday.” (BP1)*

Participants unanimously agreed that honesty was appreciated when delivering the diagnosis, even if it was extremely difficult news to cope with. This honesty, participants said, would allow parents to have an open conversation about the potential next steps, i.e. treatment options, with their physician.

*“It’s that human side of the doctors, and still that professional side of having to deliver the information.” (BP1)*

Along with honesty, parents of children currently in treatment highlighted that oncologists use medical jargon during the initial diagnostic meeting, which exacerbates the issue of understanding and/or accepting the diagnosis:

*“I had no idea what [the diagnosis] was [ … ] I didn’t even know [my child] had cancer until they presented us with this clinical trial and as my mom was reading the drugs, she’s like … well that’s chemotherapy. And then, I’m like … [my child] has cancer?” (PT3)*

These parents agreed that physicians tend to ‘protect’ parents from the truth by not saying the word ‘cancer’ or refraining from referring to the treatment as “chemotherapy:”

*“I kept telling people, oh no, my son doesn’t have cancer. It’s just a low grade glioma. He doesn’t have cancer [ … ] and then the Canadian Cancer society sent us a letter saying [my child] is registered as a cancer patient. Oh okay – maybe it’s cancer then [ … ] but I haven’t heard [cancer] from a doctor. [...] The [doctors] try to help us to cope, you know. But sometimes it’s better you give us some straight information.” (PT4)*

Nevertheless, bereaved parents acknowledged how challenging it must be for the oncologists, since they are given the difficult task of delivering the results and prognosis in a realistic and honest manner, without giving false hope to the parents:

*“But thank goodness, most of them are honest, and that’s what we needed. And I think that’s important for physicians and oncologists to know that.” (BP8)*

Conversely, parents of children in treatment highlighted how negative results could take away hope for a cure and this will be difficult for the parents to accept. They suggested that physicians acknowledge the uncertainty around the accuracy of the LDT, as no test is 100% accurate, and that many parents will try new therapies, even if the child may not respond to them:

*“If your child is in the 1% that doesn’t respond to anything, you’re taking away their hope. And the human spirit and hope is a huge factor. If you believe in something and have faith that something is going to work, sometimes [it works].” (PT1)*

Moreover, parents of children in treatment also focused their discussion on how the information divulged in the first clinical encounter can be overwhelming; they suggested that physicians schedule multiple meetings with the parents, where the information can be delivered in measured doses and important concepts can be repeated at each meeting. The gap between successive meetings would allow the parents to process the information and return to the next meeting with questions. One participant recalls:

*“It took a while to register. It took at least 4 meetings with [our doctor] before it got into our heads that there is nothing [they can do].” (PT4)*

They also suggested that treatment decisions should not be made at the initial diagnostic encounter, given that parents typically have trouble understanding and/or accepting the diagnosis.

In the event that the genetic test suggests the tumor would not respond to any available therapies, parents first discussed what type of information should be presented when disclosing a catastrophic diagnosis and the support the care team should have at the initial diagnostic consultation, including from the palliative care team. First, they highlighted that physicians should expect parents to request a second and/or third medical opinion to reduce uncertainty of the prognosis, regardless of the expected accuracy of the test; this was particularly important if the parents had not built a trusting relationship with the treating oncologist. Second, participants agreed that physicians should provide supporting evidence for the diagnosis from other tests such as an MRI to help parents better understand and accept their child’s prognosis. Finally, participants recommended presenting statistics of the tumor type and why this specific tumor would not respond to chemotherapy to parents during the initial diagnostic consultation:

*“If you have more pieces [of information] that you can put together later when you’re thinking about the [encounter] then it’s a little bit easier.” (BP5)*

In addition to this, parents of children currently in treatment recommended that the child not be present during the initial diagnostic meeting, as the parents would need time to fully understand and accept the diagnosis before being able to explain it to their child.

With regard to support, parents recommended having an assigned social worker, bereaved parent, general practitioner who has built trust with the family, or other psychological support personnel in the room at the initial diagnosis or when a relapse occurs who can relate to what the parents are going through as well as help them navigate the process:

*“You need someone who knows what has been happening [ … ] almost like a bereaved parent, who can guide you through it.” (BP8)*

However, parents of children currently in treatment also mentioned that too many people in the room could also be intimidating:

“*The first time, we were just sitting in the waiting room, and this guy comes in … we’ve never seen him before and says, your son has a brain tumor. That’s how we found out. I didn’t even know if he was a doctor [ … ] but then the second time, we had all these people and I thought … Oh no, it must be bad. Because the first time, it was the janitor who told me and now we have the social workers here so it must be worse.” (PT2)*

Participants also suggested having someone who can take notes during the initial diagnostic encounter with the treating physician, summarizing important points and outlining the potential treatment options:

*“Maybe it’s the social worker or someone else who comes in to take notes [ … ] because you’re listening but you’re not really listening. As soon as the diagnosis comes in, it’s like bla bla bla, the teacher from Charlie Brown.” (BP1)*

Bereaved parents were also concerned about the anxiety and worry that may result from the association of the word “DNA” or “genomic” to the inheritance of the brain tumor. Participants agreed that this may cause parents to feel as though they were to blame for their child’s tumor and that their other children may be at risk of acquiring this deadly form of cancer:

*“If you can test for this, then I want my other children tested … because if it’s in the DNA, they would see that as a whole family thing as I can imagine [ … ] I would just sit there and think … is this going to happen to my other child?” (BP6)*

Bereaved parents agreed that it would also be important to provide psychological support to the siblings of the diagnosed child, as they may worry about eventually developing the disease:

*“I’m sure it’s in the back of [my other son’s] mind … what if this is genetic and I have it?” (BP4)*

Whether and when the palliative care team should be introduced was a subject of deliberation amongst bereaved parents. Initially, they disagreed about the timing of the introduction; some felt that the palliative care team should be present at the initial diagnostic encounter, while others felt overwhelmed by the ‘mob of physicians’:

*“It’s almost like they were all there to protect themselves from us freaking out on them. I don’t know. I felt a little up against the wall with so many people being there delivering that news.”(BP8)*

After further discussion, bereaved parents agreed that the palliative care team and other health care professionals should be introduced to the family early on, but not at the initial diagnostic consultation.

Once the prognosis has been communicated, all parents recommended that oncologists hold another meeting with the family to discuss potential treatment options as well as the risks and benefits of each option since it was widely felt “the more knowledge, the better.” Parents discussed how the options should be presented, the burden of making the treatment decision, and how the LDT could improve the quality of life of children with intractable brain tumors by not pursuing highly toxic therapies known to be ineffective.

Regardless of the test results, parents valued honesty from the health professional as well as trust; it is this honesty and trust built with the continuity of care received by the treating physician that allows parents to accept the dismal prognosis and eventually guide treatment decisions that maximize their child’s quality of life:

*“I mean, that’s one of the biggest problems too, right, is that you're always seeing a different doctor [ … ] And if one of them brought this new test, to give this … And you didn’t know that doctor, and you hadn’t seen them much before, and you didn’t quite like them [ … ] to trust that this test was useful or that you would want to do it [ … ] But they all seem to work that way now. You don’t see the same doctor. It’s always somebody else.” (PT3)*

Some participants also highlighted how important it is for the treating physician to be in the know, as many of the parents will base treatment decisions on their physician’s recommendations and trust that they will provide them with accurate, honest, and unbiased information to make this decision:

*“We needed someone to say to us that chances are, even if it does work, [your child] will probably not last another year … So how do you want that year to go? A lot depends on what the physicians are telling you.” (BP8)*

All parents agreed that the treating physicians should explain all possible treatment options, including clinical trial enrolment, to the parents without persuading them toward one option or another. It should be up to the parents and child, where appropriate, to make the final treatment decision:

*“It’s going to be tough for the physicians to give the right “answer” [regarding treatment options]. They gave us both sides of the coin [ … ] Ultimately, it’s a chance of a chance, but you as parents are the ones that have to make that decision. And I think that’s tough for a lot of parents to hear. Even though they are not the professional, they will still have to make that choice at some point. Our care was amazing [ … ] it was always that honest, up front, there isn’t hope but we’ll help you if you want to look at other things … We’ll help you if you want to do these trials, but we’re also here to make [your child’s] life as comfortable as possible for what we think is left of it.” (BP1)*

Parents of children in treatment also discussed when the child should be included in treatment conversations with the oncologist. They recommended that the oncologists initially speak to the parents about the treatments and then have the parents choose what information is relevant to the child so as to avoid giving them information that may lead to unnecessary worry. However, parents agreed that children should be included in decision-making:

*“You have your discussion with the doctor and then bring [your child] in after and say,[ … ] we’ve been discussing different [treatment] options and what we think is best for you is this. And what do you think? Do you have any questions?” (PT1)*

With respect to delivering news about the failure of treatment, parents suggested that the care team approach the delivery of such information in a coordinated way, i.e. the health care team does not send mixed messages about the prognosis to the family, so as to avoid any unnecessary confusion or distress:

*“One doctor came in and said, we’ve got bad news, the drug didn’t work and you guys are going to go into radiation. Then he left and another doctor comes in and says, oh we have wonderful news, we’re going to start the radiation soon. One doctor is telling you one thing, and the other says wonderful news. Which one is it?” (PT4)*

Lastly, parents also agreed the LDT has the potential to significantly improve the quality of life of children with HGGs not responsive to chemotherapy. Non-invasive treatment, i.e. radiation, was viewed favorably in terms of improving quality of life since the side effects were minimal and symptom management was critical. In contrast, chemotherapy was seen as more invasive and many bereaved parents expressed their regret for deciding to follow this treatment path having known retrospectively of its inefficacy in treating their child’s specific tumor:

*“The worst part was chemotherapy. [ … ] At that time you think you’re doing the right thing, because that’s what they are telling you to do [ … ] They’re sick, then they’re skinny, then they’re fat, then they’re skinny, then they’re fat. Thinking back on it now, I wish I would have just taken him home and enjoyed my time with him.” (BP3)*

### Mixed consultation

The mixed consultation ratified many of the issues highlighted in Phase I small-group deliberative consultations and led to six important family-centered communication recommendations for the implementation of the LDT in clinical practice. First, the risks, benefits, and accuracy of this non-invasive blood test, highlighting that it avoids brain biopsies to confirm diagnosis and can also inform treatment strategies, should be clearly communicated to parents at the initial encounter. Second, to address issues with medical jargon, disjointed communication in the medical team, as well as overwhelming parents with too much information that they will likely not remember or understand, a lay written summary of the main points, including a summary and interpretation of the LDT and other test results, from consultations with the care team should be provided to families to further improve communication, understanding, and acceptance of the diagnosis and prognosis of the child. Third, oncologists should pre-plan several successive meetings with the families to ensure that they provide detailed and clear information to parents in measured doses without overwhelming them during the initial encounter, which is particularly crucial when the diagnosis is catastrophic. Fourth, all families should be provided with a standardized protocol for psychosocial support, including access to social workers, a parent who has gone through a similar situation that can help them navigate the system, or other counselling support programs that may exist in the hospital. In the event that the diagnosis is catastrophic, the palliative care team should be introduced early on in the process rather than later. Fifth, with respect to how the treating physician should approach communication of the diagnosis and/or prognosis to the family, honesty, empathy, and clarity were stressed as central values to avoid causing families unnecessary confusion and distress. Last, training in compassionate and empathetic communication of medical information, including diagnostic, treatment, and prognostic information, should be provided to medical students, residents, and physicians in pediatric oncology.

## Discussion

The deliberative consultations with bereaved parents and those of children currently undergoing treatment for difficult-to-treat brain cancer highlighted communication needs between the clinical care team and families, and led to six concrete recommendations for LDT-based communication to parents in this context. Moreover, the salient themes in our deliberations align with five of six core functions of family-centered communication, as outlined by a systematic review conducted by Sisk et al.: fostering healing relationships, exchanging information, responding to emotions, making decisions, and managing uncertainty [14].

Our finding that, to foster healing relationships, prognostication, where catastrophic or not, should be communicated in an honest, empathic, and compassionate way, was consistent with the literature [3, 15–17]. Parents expressed how unclear or withheld information led to heightened distress and confusion, which have both been shown to affect trust in the treating physician [18] and the clinical information conveyed to them [19].

How and what diagnostic, prognostic, and treatment information should be exchanged was also addressed in all deliberations. Consistent with other studies [3, 17, 20, 21], parents stressed the importance of providing detailed, clear, and unbiased information as well as supporting evidence for the diagnosis. Parents cautioned, however, about being overwhelmed at the initial diagnostic meeting, recommending that information be delivered and repeated on an ongoing basis in subsequent meetings. This recommendation is consistent with several previous studies highlighting the ongoing information needs of parents of children with cancer [15, 17, 19, 22–26]. Moreover, parents of children currently undergoing treatment for difficult-to-treat brain tumors deliberated on the direct involvement of their children when information is exchanged, suggesting that discussions first take place with parents and then with their children. While some previous studies showed variation in parents’ preferences for the direct involvement of children in discussions with the physician [15, 27], others have also found that parents preferred to first process the information separately from their children [24, 28, 29].

Avoiding the word ‘cancer’ and “chemotherapy” or using vague medical jargon were identified by some parents as possible coping mechanisms used by oncologists to protect themselves when delivering bad news. Honesty and empathy were re-iterated as crucial communication components of emotional support, as in other studies [28, 30], allowing parents to react and eventually accept the diagnosis. Parents named emotional support personnel, including social workers, parent guides, counsellors, or the palliative care team (for a catastrophic diagnosis) as especially key to helping them navigate their new reality, and recommended such services be offered to all families regardless of the prognosis. In the event of a catastrophic diagnosis, when the palliative care team should be introduced at the initial diagnostic meeting was a point of disagreement between the bereaved and treatment parent groups, although both groups acknowledged that they should be introduced early on in the process. This introduction rarely occured early for many parents, which could demonstrate a potential divide between palliative care and oncology teams [31]. Parents also expressed a need for emotionally supporting siblings of affected children, due to the links between genetics and the type of brain cancer, which, to our knowledge, has not yet been described in previous studies.

When it comes to decision-making, parents preferred to be engaged and presented with all treatment options, both in terms of benefits and risk, in a clear and unbiased way, i.e. without persuasion into a clinical trial [3, 17, 32]. In terms of the risk-benefit of each treatment option, parents also requested information of the treatment’s impact on the child’s quality of life. Allowing time in between the first diagnostic meeting and subsequent meetings where treatment options are explored was also viewed favorably [20]. These communication strategies can foster trust, understanding, and peace-of-mind, making parents feel supported during the decision-making process.

Lastly, uncertainty around the diagnosis was a salient theme in all deliberations. Regardless of a test’s demonstrated accuracy, parents expressed that they will always question a result suggesting poor or catastrophic diagnosis. Acknowledging the margin of error around the information presented to parents was deemed important in discussions and parents suggested that physicians be supportive of a request for a second or third opinion to reduce even the slightest uncertainty.

Our study corroborates many findings from the cancer communication literature, but is novel in at least two primary ways. First, it is among the first to apply deliberative consultation methods to develop family-centered communication recommendations, and second, to explore communication needs ahead of clinical translation and implementation of a pharmacogenomic test in the pediatric cancer clinic. These methods not only fostered a feeling of peer-support between members of each deliberation, but also led to a trusting research partnership with Meagan’s Walk. We did not recruit a representative random sample of bereaved parents or parents of children currently undergoing treatment for brain cancer, which is a potential limitation of the study. The themes highlighted in our deliberations are, however, consistent with those reported across other pediatric cancer contexts and lends robustness to this method of engagement.

## Conclusion

Our findings have direct implications for implementing the LDT in clinical practice. The agreements reached by parents in the mixed deliberation provide key family-centered recommendations for communicating results and informing clinical decision-making. Among the recommendations, lay written summaries of the information disclosed in each meeting with the clinical care team as well as access to emotional support personnel, including genetic counselling for the family, should be offered alongside the implementation of the LDT. Standardized training in sensitive and honest communication should be offered to medical students, residents, and physicians in pediatric oncology to better equip them when faced with the challenge of disclosing poor diagnoses. Lastly, methods of engagement, such as deliberative consultations, can be important tools in the implementation plan of novel diagnostic tests in pediatric cancer.

## Supplementary Information

**Additional file 1.**

## Data Availability

The datasets generated and/or analysed during the current study are not available due to privacy and confidentiality of the statements made by deliberants in documented transcripts.

## Abbreviations

HGG: High-Grade Glioblastoma
LDT: Laboratory Derived Test
