# Supplementary material for: Communication of Pharmacogenomic test results and treatment plans in pediatric oncology: deliberative stakeholder consultations with parents
Source: BMC Palliat Care. 2021 Jan 12;20:15. doi: 10.1186/s12904-021-00709-2 (PMC7805194; doi:10.1186/s12904-021-00709-2)
Supplement: Supplementary file 1 — Additional file 1. [file 12904_2021_709_MOESM1_ESM.docx]

**Supplementary Materials 1.** Deliberative democratic methods allow researchers to unveil normative values specific to a forthcoming policy from the perspectives of key stakeholder groups, in this case parents, as well as make practical recommendations for the policy’s implementation (Rychetnik, 2015). The substantive outputs of deliberation notwithstanding, the quality of the deliberative process is important to ensuring these outputs are the result of democratic, and participatory processes from interested stakeholder. Put simply, “if the deliberative process is to be trusted, we need to know more about what happens as people deliberate*”*(De Vries et al., 2010)*.* In addition to reporting on the substantive outputs, we also assessed the deliberative quality of three stakeholder consultations by administering two, post-consultation evaluation surveys adapted from DeVries (De Vries, Stanczyk, Ryan, & Kim, 2011).

An expert facilitator gave an informational presentation to parents at the start of the deliberation, which covered background evidence on the current i) incidence, prevalence and burden of disease of pediatric high-grade glioma; ii) mortality data for specific HGG variants; and iii) sensitivity and specificity of a newly developed LDT to detect HGG variants. Parents were invited to ask the facilitator any clarifying questions in response to the information presented and prior to launching an open discussion concerning implementation of the new LDT. An evaluation of deliberative quality survey, and an information assessment measure (IAM) were used to assess the deliberation from *process* and *informational* elements according to the DeVries et al framework. Parents rated the quality of their deliberative experience, and self-reported on what they learned using a 10-point Likert scale (1 being not all and 10 being very much). Descriptive statistics, including group means and ranges were then calculated from these scores. Results from the evaluation of deliberation survey (**Table 1**) and the IAM (**Table 2**) are presented below.

Parents across the three groups reported feeling listened to (average 10/10) and respected (average 9.9/10) throughout the deliberation process. These averages were likewise high in response to survey items related to procedural fairness and helpfulness of the information presented. One parent noted in the open-ended response field that the session was especially therapeutic for them, and expressed how lonely and all-encompassing this diagnosis can be for families with few opportunities to share their experience with others. Results from the IAM corroborated the informational value reported in the evaluation survey. The majority of deliberants reported they learned something new and were motivated to learn more when asked in what ways the information impacted them (**Table 2**).

In general, parents did not change their opinions on the use and implementation of the LDT after attending session. However, given that disagreements in all consultations were uncommon, this finding is not surprising. While not a detriment to deliberative quality per se, this finding is noteworthy to consider in light of the implementation recommendations which resulted from the study. For example results from the IAM suggest parents perceived some benefit to implementing the LDT as none reported feeling dissatisfied, alarmed or thought the information was potentially be harmful.

**Table 1**. Results of post-consultation evaluation surveys from bereaved parents and parents whose children were currently undergoing treatment for HGG (N = 12). Survey items adapted from the DeVries et al framework for assessing deliberative quality using a 10-point Likert scale (1 being not all and 10 being very much).

|  | **Bereaved** | **In treatment** | **Mixed** | **Comments** |
| --- | --- | --- | --- | --- |
| Do you feel that your opinions were respected by your group | 9.8 | 10 | 10 |  |
| Do you feel you were listened to by your group | 10 | 10 | 10 |  |
| Do you feel that the process that led to your group's response was fair? | 8.75 | 10 | 10 |  |
| How willing are you to abide by the group's final position, even if you personally have a different view? | 7.1 | 10 | 9.25 |  |
| How helpful did you find a) question and answer interaction with the experts? | 9.75 | 9.75 | 10 |  |
| How helpful did you find b) the formal presentations given by the experts | 9.25 | 10 | 10 |  |
| How helpful did you find c) discussing the issues with other participants? | 9.5 | 10 | 10 | Very useful session and very therapeutic. Often, we have no one to talk to but brain cancer has affected all of our beings and all of our families. |
| How much did attending the session change your understanding about the use of this new pharmacogenomics test in pediatric oncology? | 9.38 | 8.5 | 7.6 |  |
| How much did attending the session change your opinion about the use of this new pharmacogenomics test in pediatric oncology? | 8.25 | 8 | 5 |  |
| How useful was the session in helping you answer the survey questions? | 9.25 | 9.5 | 9.5 |  |

**Table 2**. Results of the information assessment measure (IAM) survey from bereaved parents, parents whose children are currently undergoing treatment for HGG and a mixed group of parents from both the aforementioned groups (N = 12).

|  | I learned something new | I'm motived to learn more | This information confirmed I did (am doing) the right thing | I am reassured | I am reminded for something I already knew | I am dissatisfied | There is a problem with the presentation of this info | I disagree with the content of this info | This information is potentially harmful |
| --- | --- | --- | --- | --- | --- | --- | --- | --- | --- |
| **Bereaved** | 6 | 6 | 4 | 1 | 1 | 0 | 0 | 0 | 0 |
| **In treatment** | 4 | 4 | 3 | 3 | 1 | 0 | 0 | 0 | 0 |

De Vries, R., Stanczyk, A. E., Ryan, K. a., & Kim, S. Y. H. (2011). A Framework for Assessing the Quality of Democratic Deliberation: Enhancing Deliberation as a Tool For Bioethics. *Journal of Empirical Research on Human Research Ethics: An International Journal*, *6*(3), 3–17. https://doi.org/10.1525/jer.2011.6.3.3

De Vries, R., Stanczyk, A., Wall, I. F., Uhlmann, R., Damschroder, L. J., & Kim, S. Y. (2010). Assessing the quality of democratic deliberation: A case study of public deliberation on the ethics of surrogate consent for research. *Social Science & Medicine*, *70*(12), 1896–1903. https://doi.org/10.1016/j.socscimed.2010.02.031

Rychetnik, L. (2015). Which public and why deliberate ? - a scoping review of public deliberation in public health and health policy. *Social Science & Medicine*, *131*, 114–121.
